# Playing ‘Pong’ Together: Emergent Coordination in a Doubles Interception Task

**DOI:** 10.3389/fpsyg.2016.01910

**Published:** 2016-12-06

**Authors:** Niek H. Benerink, Frank T. J. M. Zaal, Remy Casanova, Nathalie Bonnardel, Reinoud J. Bootsma

**Affiliations:** ^1^Institut des Sciences du Mouvement, Aix-Marseille Université, CNRSMarseille, France; ^2^PsyCLE, Aix-Marseille UniversitéAix-en-Provence, France; ^3^Center for Human Movement Sciences, University Medical Center Groningen, University of GroningenGroningen, Netherlands

**Keywords:** joint-action, coordination, decision-making, collaboration, interpersonal coordination, perception-action, team, interception

## Abstract

In this contribution we set out to study how a team of two players coordinated their actions so as to intercept an approaching ball. Adopting a doubles-pong task, six teams of two participants each intercepted balls moving downward across a screen toward an interception axis by laterally displacing participant-controlled on-screen paddles. With collisions between paddles resulting in unsuccessful interception, on each trial participants had to decide amongst them who would intercept the ball and who would not. In the absence of possibilities for overt communication, such team decisions were informed exclusively by the visual information provided on the screen. Results demonstrated that collisions were rare and that 91.3 ± 3.4% of all balls were intercepted. While all teams demonstrated a global division of interception space, boundaries between interception domains were fuzzy and could moreover be shifted away from the center of the screen. Balls arriving between the participants’ initial paddle positions often gave rise to both participants initiating an interception movement, requiring one of the participants to abandon the interception attempt at some point so as to allow the other participant to intercept the ball. A simulation of on-the-fly decision making of who intercepted the ball based on a measure capturing the triangular relations between the two paddles and the ball allowed the qualitative aspects of the pattern of observed results to be reproduced, including the timing of abandoning. Overall, the results thus suggest that decisions regarding who intercepts the ball emerge from between-participant interactions.

## Introduction

Actions in our daily life often involve others. Whether we are shaking someone’s hand, moving a table together or walking on a crowded pavement, we have to coordinate our actions with those of other individuals. Such social coordination, whether it is intentional or spontaneous, often requires decisions about the behavior that we should perform or, in some cases, we should *not* perform. For instance, safe driving dictates that when two drivers simultaneously approach an intersection one should cross first and the other should wait. Likewise, two individuals loading a dishwasher should take their turns when placing the dishes. Besides interacting with one another, these situations typically demand a decision of who performs an action and who does not. It is such joint decision making among individuals in goal-directed joint activities that we address in the present study. To do so, we started from a pertinent example in a sports context: serve reception in beach volleyball. When facing a serve, only one of the two players of a team should perform the actual serve reception. The non-receiving player should not interfere during the interceptive action of the teammate, while, at the same time, preparing a follow-up action. How do such individuals coordinate their actions and decide who will intercept the ball? In this contribution, we captured the essential characteristics of the beach volleyball situation in a task in which two participants play “doubles pong.” The participants’ task was to ensure that on each trial one of them intercepted the approaching target. Like in the situation of serve reception in beach volleyball, the players have to decide together who will be the one performing the interceptive action (and who will not). We are interested in the way the decision of ‘who intercepts the balls where’ is shaped and how such joint decision making may best be captured.

Rather than focusing on the neural processes that are involved in decision making within each individual (e.g., Cannon-Bowers et al., 1993; DeSoto et al., 2001; Bogacz et al., 2006; Cisek, 2007; Resulaj et al., 2009; Lepora and Pezzulo, 2015), here we consider the system of the two individuals and their environment (cf. Araújo et al., 2006; Marsh et al., 2006; Richardson et al., 2010; Theiner et al., 2010; Riley et al., 2011; Schmidt et al., 2011; Coey et al., 2012). We study how the coordinated behavior of this system gives rise to a distribution over the individuals of interception activities. Instead of understanding decisions as mental operations that precede action, we see the act of deciding as the emergent behavior of the system of the individuals and environment resulting in (un)successful task performance (cf. Turvey and Shaw, 1995; Araújo et al., 2006; Travassos et al., 2012; Barsingerhorn et al., 2013). Understanding decision making among individuals as emergent is in line with a dynamic-systems approach initially developed to account for intrapersonal coordination of rhythmic movements (e.g., Haken et al., 1985; Kugler and Turvey, 1987). From a dynamic-systems perspective on human movement the goal is to identify general laws and patterns that govern the causal unfolding of a system’s behavior rather than looking for neurophysiological areas that generate behavior (Kelso, 1995). Importantly, the stability principles underlying the emergence of coordination in a system of coupled oscillators have been demonstrated to operate whether the coupling is neural (Kelso et al., 1981), mechanical (Bardy et al., 1999, 2002) or informational (Schmidt et al., 1990; Schmidt and O’Brien, 1997; Richardson et al., 2007). That is to say, the same phenomena related with stability of patterns are found when a single person coordinates two body parts and when two persons contribute one body part each to the coordination (Schmidt and O’Brien, 1997; Richardson et al., 2007; see Schmidt and Richardson, 2008, for a review). This similitude principle indicates that the dynamic-systems approach can account for interactions at different behavioral levels, independent of the nature of the connections between the system’s components (i.e., neural, mechanical or informational). Whereas most of the studies addressing the dynamics of joint actions concerned non-functional or stereotyped oscillatory limb or whole-body movements (such as swinging legs or rocking chairs together, Schmidt et al., 1990; Richardson et al., 2007), a few studies have shown that the interactive behavior of two individuals can also account for the observed coordinated patterns in more goal-directed tasks (Mottet et al., 2001; Richardson et al., 2015; Romero et al., 2015).

The shared goal of the players in a beach-volleyball situation is that the approaching serve will be intercepted by one of the two. In order to understand the dynamics of joint decision-making in such a cooperative goal-directed interception task, in the doubles-pong task adopted here we explored how a team’s task performance might emerge from the interactions between participants. For the present purposes, potential interactions in this video-game-like task were restricted to be uniquely information-based: without any other form of communication being available, participants only shared vision of the task space (i.e., screen) in which the target and individual participant-controlled interception paddles moved. With each of the two paddles being moreover confined to one-dimensional movement along a common interception axis, the task design ensured that successful interception could only be achieved by a single participant: contact between the two paddles immediately eliminated all future possibilities for interception. Because the task of the team of players involves the interception of the ball by one of them, and this lateral interception closely resembles tasks that have been studied extensively before (e.g., Peper et al., 1994; Michaels et al., 2006; Ledouit et al., 2013; Bootsma et al., 2016), we expect that the current study might serve as a stepping stone for identifying informational variables that may underlie team behavior.

## Materials and Methods

### Participants

A group of 12 right-handed students from the University of Aix-Marseille, eight men and four women with an average age of 19.6 ± 1.0 years (*M* ± *SD*), took part in the experiment. They all provided written consent before participating voluntarily in our study. The study was approved by the local institutional review board of the Institute of Movement Sciences (*Comité Ethique de l’Institut des Sciences du Mouvement d’Aix-Marseille Université*) and conducted according to University regulations and the Declaration of Helsinki.

### Task

**FIGURE 1:**
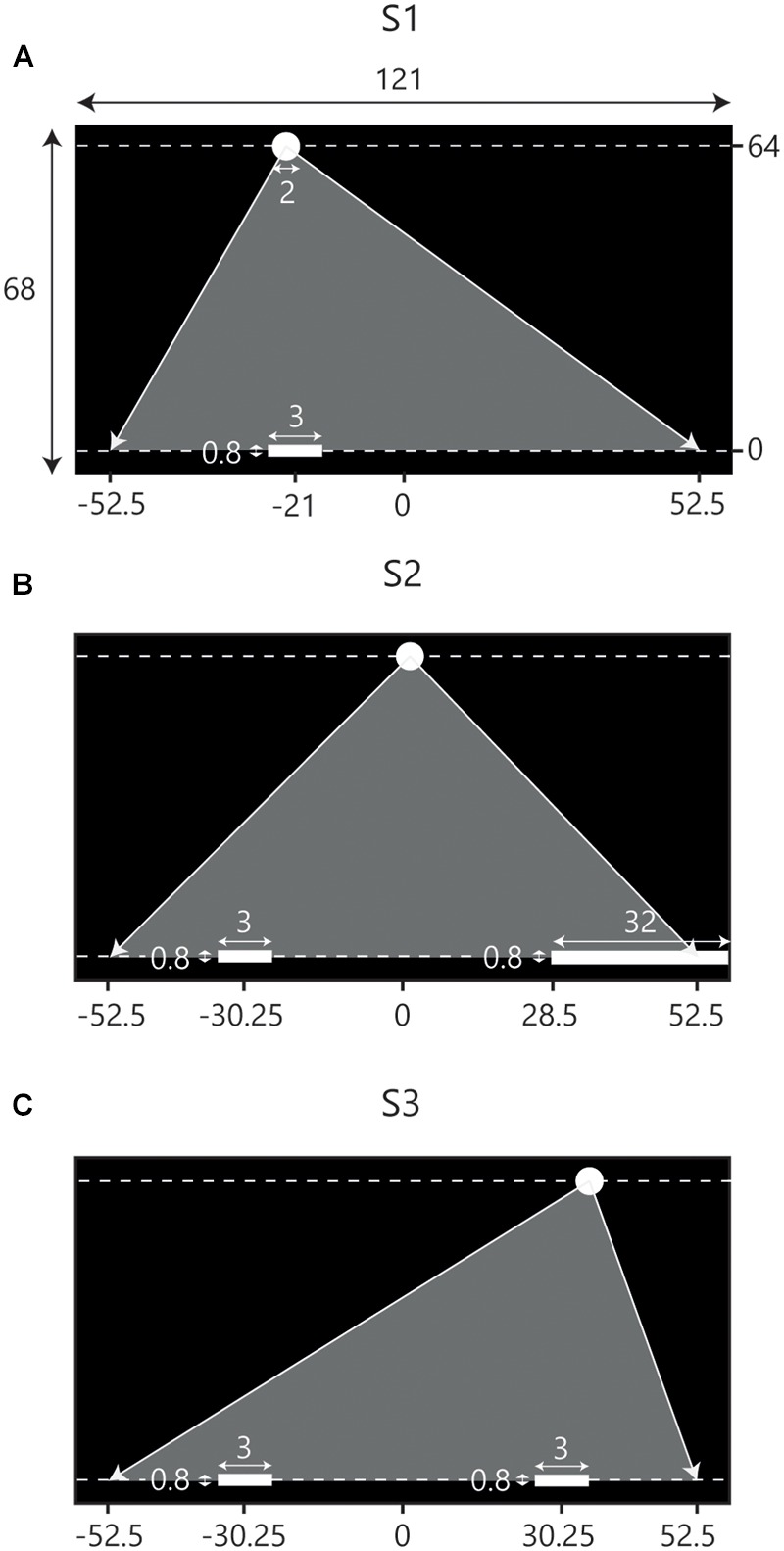
**Schematic overview of the set-up of the three consecutive experimental sessions.** Screen dimensions and other metrics are in cm. Note that the figures are not scaled to actual size. Balls appeared at the top of the screen (*Y* = 64) and moved downward toward the interceptions axis (*Y* = 0) at one of two constant vertical velocities. Gray triangles indicate the range of potential ball arrival positions. **(A)** During the first session (S1) participants intercepted balls individually. The situation depicted here represents the initial conditions for LP. **(B)** In the second session (S2) participants were assisted by a stationary partner, incorporated by a static paddle covering the final 24 cm of the range of potential ball arrival positions on the opposite side of the interception axis. The situation depicted represents the initial conditions for LP. **(C)** During the third session (S3) participants intercepted balls in dyads where LP started on the left side of the screen and RP started on the right side of the screen.

The experiment consisted of three consecutive sessions in which participants were to manually intercept virtual balls moving downward across a screen. A ball could be intercepted by moving an on-screen paddle laterally over an invisible horizontal interception axis at the bottom of the screen. During the first experimental session participants intercepted balls individually (**Figure 1A**). This session served to familiarize participants with the experimental set-up. In addition, by counting the number of intercepted balls, we obtained a measure of how well individual participants performed the interception task. The second experimental session was, again, an individual-participant session. This time, however, participants were assisted by a static “partner” incorporated by a large stationary paddle located at the opposite side of the interception axis (**Figure 1B**). Balls arriving at the stationary paddle were returned upward and counted as a successful interception. Participants had to avoid touching the static paddle; on-screen contact immediately led both paddles to disintegrate and interception was no longer possible. In the third experimental session participants performed the interception task in teams (**Figure 1C**). We composed teams of two participants with similar interception scores on the first two sessions. Like in Sessions 1 and 2, participants were able to move all along the interception axis and, comparable with Session 2, they should avoid touching one another; both paddles would disintegrate if they did. No communication in any form was allowed.

### Experimental Set-Up

**FIGURE 2:**
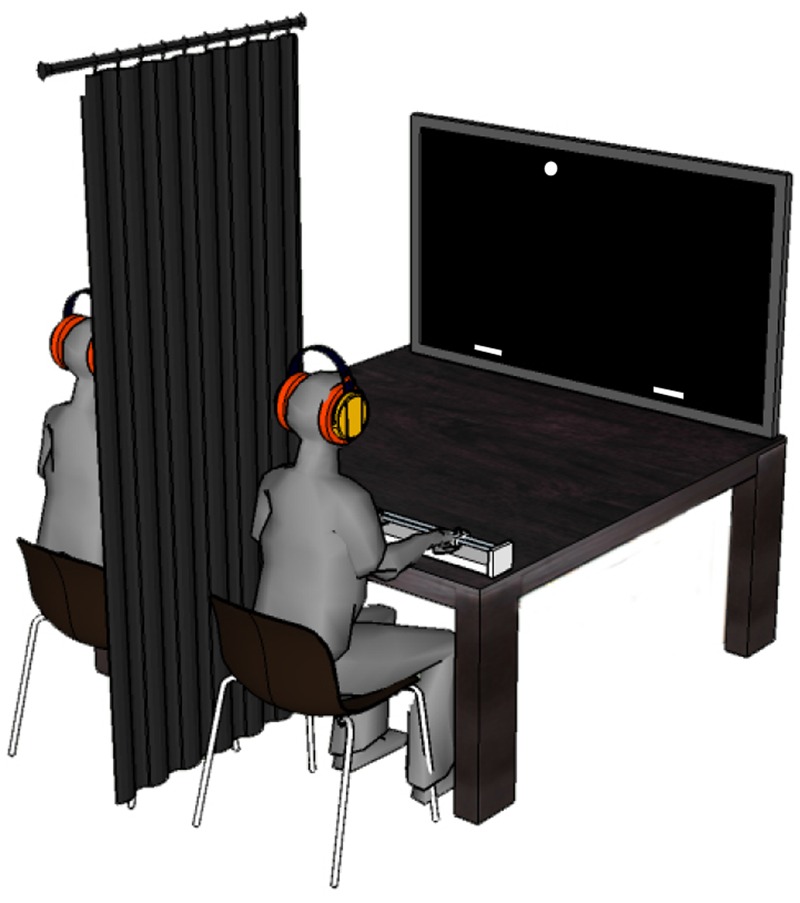
**Representation of the experimental setting used in Session 3.** Participants were sitting side by side facing a large television screen. They were separated by a black curtain and wore headphones and earplugs so as to avoid overt communication between them. To intercept the balls moving downward across the screen, both participants could move an on-screen paddle along the (non-visible) interception axis by displacing a handheld knob on a linear positioning device placed on the table in front of them. In Sessions 1 and 2 only one of the participants was present.

The experiment took place in a darkened room without windows. **Figure 2** present the experimental setting for the session in which two participants performed the task together. Participants were seated at one of the two possible seats on one end of a table. They were facing a large television screen (Samsung 55″ LED ED55C, with a 1920 × 1080 pixels resolution) that was positioned 2 m away at the other end of the table. When seated, the participants faced the screen at eye height. Six participants were always seated at the right side of the table during each of the three sessions and are referred to as Right-side Participants (RPs); the other six participants always sat left and are referred to as Left-side Participants (LPs).

Using their right hand, participants displaced the on-screen paddle by laterally displacing a handheld knob on top of an in-house-constructed linear positioning device placed on the table in front of them. The knob was firmly attached to a small (3 by 6 cm) aluminum cart that could slide along two (75-cm long) parallel iron bars. The cart’s position was sampled at a frequency of 100 Hz using a linear magnetic potentiometer (MP1-L-0750-203-5%-ST, Spectra Symbol, West Valley City, UT, USA) connected to the computer (HP ZBook 15) controlling the experiment. The digitally sampled electrical output of the potentiometer was converted by in-house developed ICE^®^ (ISM, Aix-Marseille Université, France) software into a paddle position using a constant gain, such that the two extreme knob positions corresponded to (virtual) screen positions slightly beyond the physical screen. This allowed participants to cover the full (121 cm) length of the interception axis on the screen without ever reaching the extremities of the 75-cm long positioning device. Unless specified otherwise, positions and distances reported from here on correspond to distances on the screen, with the origin corresponding to the horizontal center of the interception axis. The screen thus extended horizontally (*X*-axis) from -60.5 cm to +60.5 cm and vertically (*Y*-axis) from -2 cm to +66 cm.

### Procedure

Participants had to intercept virtual (2-cm diameter circles) white balls depicted against a black background, moving downward across the screen at various angles and speeds, by making them bounce back upward after contact with their white (3-cm wide and 0.8-cm high) paddle.

For a trial to start, participants moved the paddle to a designated start position (see **Figure 1**) positioned at ±21 cm from the center of the screen in Session 1 and at ±30.25 cm from the center of the screen in Sessions 2 and 3. Start positions were marked by a 3-cm wide translucent red rectangle that would turn green when the center of the paddle was located at a horizontal distance of less than 0.3 cm from the center of the rectangle. After the participant(s) had remained in place for 2 s, the rectangle disappeared and after another 2 s the appearance of a ball at the top of the screen marked the beginning of the trial. Balls immediately moved downward with vertical velocities of 0.40 or 0.64 m/s corresponding to movement durations until reaching the interception axis of 1.6 and 1.0 s, respectively.

Ball trajectories were constructed with the use of five standard ball departure positions (*Y* = 64 cm) and five standard arrival positions (*Y* = 0 cm), both at *X* = -42, -21, 0, +21 and +42 cm. Combining the five departure positions with the five arrival positions gave rise to a total of 25 standard rectilinear trajectories. To avoid participants becoming familiarized with the arrival positions of the ball, a random distance between -10.5 cm and +10.5 cm was added to both the standard departure and arrival positions of a trajectory. This way, balls could appear and arrive everywhere between *X* = -52.5 cm and *X* = +52.5 cm while trajectory angles were kept the same. In a single block, all 25 trajectories appeared with two different vertical ball velocities, for a total of 50 fully randomized trials per block. All participants performed four blocks per session, adding up to a total of 200 trials per participant in a 1-h session.

Successful interception required that the paddle touched the ball when it crossed the interception axis. After a successful interception, the paddle turned green and the ball moved back up. In an unsuccessful trial the ball continued moving downward and the paddle turned red. Three seconds after ball arrival at the interception axis, the paddle returned to its original white color and the translucent red triangle would appear again to indicate the start of a new trial.

All sessions started off with ten practice trials. During these practice trials participants were asked not only to intercept a number of balls but also to purposely miss a ball so they would have experienced all the possible actions and their outcomes. In Sessions 2 participants were also asked to touch the stationary paddle during a trial, so as to experience what would happen if they did during the experiment. For the proper experimental sessions participants were instructed to intercept as many balls as possible, without any further information being provided. To motivate the participants, the experiment was organized as a competition in which all participants competed anonymously.

In Session 3 participants were seated next to each other (see **Figure 2**). They were separated by a black cloth, hanging from the ceiling, that effectively prevented each participant from seeing (any part of) the other. Moreover, they wore headphones (3M Peltor Optime2) and earplugs (DEXTER Lm30215-10) so that they could not hear each other either. No communication in any form was allowed (both before and during the experiment). The participants were explicitly instructed that the number of interceptions per individual did not matter and that their performance as a team was the only thing that counted.

Kinematic data of the participants’ paddles and the ball was sampled at a frequency of 100 Hz and stored on an external disk. Along with the kinematic data, we registered trial characteristics including whether a participant intercepted the ball or not and, in Sessions 2 and 3, the time of a collision, if any. Before further analysis, the kinematic data was filtered with a second-order Butterworth filter with a cut-off frequency of 5 Hz run through twice in order to negate the phase shift (Ledouit et al., 2013).

### Dependent Measures

Interception scores were calculated per block as the percentage of balls intercepted from the total number of 50 balls presented. The score used to assemble the teams was the mean value of interception scores obtained during the first and second individual sessions.

Movement initiation time was defined as the first moment a participant crossed a velocity threshold of 3.0 cm/s provided that the participant’s movement amplitude reached at least 1 cm. Based on this criterion we determined for each individual trial whether, and if so when, a participant initiated a movement. Velocity-time series were obtained using a three-point central difference method. Peak velocity was determined as the maximum velocity reached during a movement.

**FIGURE 3:**
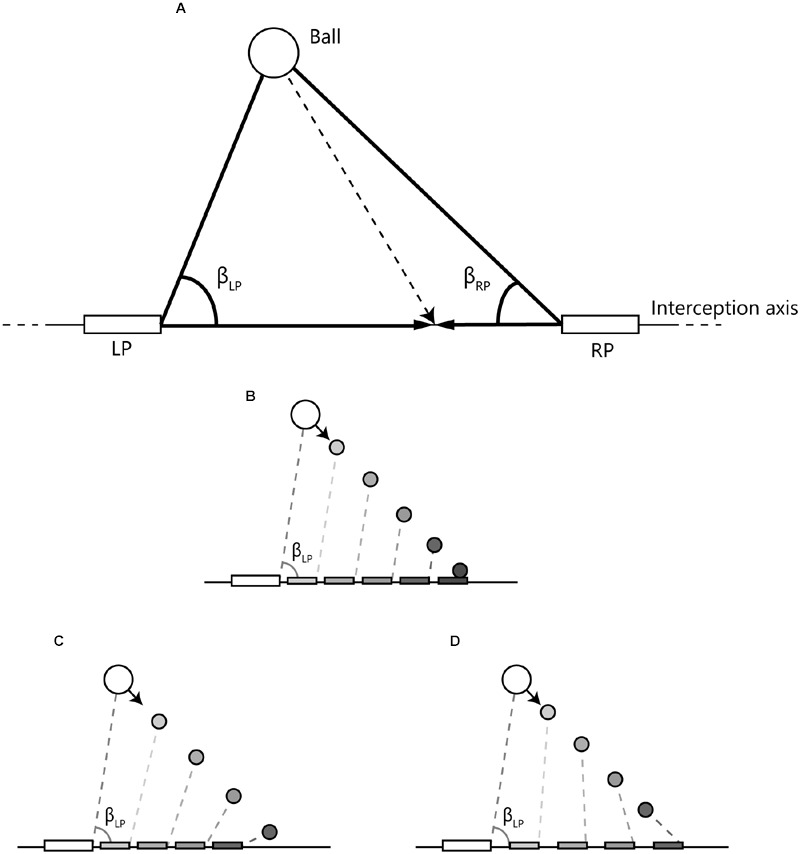
**Definition and time course examples of angles used to capture the relations between the paddles and the ball. (A)** LP and RP represent the paddles of the left and right participant, respectively, that could freely move along the interception axis. β_LP_ and β_RP_ are the angles formed by the line connecting both paddles and the lines connecting each paddle with the ball. **(B)** When the (left) paddle moves at a speed that will bring it to reach the ball arrival position when the ball (moving at constant velocity) gets there, β_LP_ is constant over time (i.e., AV is zero). **(C)** When the paddle moves at a lower speed, β_LP_ closes (decreases) over time (i.e., AV is negative). **(D)** When the paddle moves at a higher speed, β_LP_ opens (increases) over time (i.e., AV is positive).

Defining angles β_LP_ and β_RP_ according the definition provided in **Figure 3A**, allowed deriving time-series of the rates of changes of these angles (i.e., angular velocities) for the LP and the RP. As demonstrated in **Figures 3B–D**, the manner in which a participant’s paddle movement affects the pattern of change of the angle β (i.e., the state of the angular velocity, AV) is lawfully related to the future outcome of the ongoing action (also see, for instance, Fajen and Warren, 2004). As we will detail later, this prospective character of the (visual) information provided by the LP’s and RP’s angular velocities may be used to develop an account of emergent decision making.

## Results and Discussion

### Performance on the Task

We begin by examining performance on the interception task, operationalized by the percentage of balls intercepted, in each of the three experimental sessions (see **Table 1**).

**Table 1:** Interception scores of the 12 individual participants in Sessions 1 (S1) and 2 (S2) and the six teams in Session 3 (S3), together with the number of collisions observed in Sessions 2 and 3.

Team	Side	Gender	Interception score (%)			Collisions (number)	
			S1	S2	S3	S2	S3
1	LP	M	91.5	93.0	92.5	0	1
	RP	M	92.5	95.0		1	
2	LP	M	90.0	89.5	92.5	0	1
	RP	M	91.0	94.0		3	
3	LP	M	87.5	91.5	95.5	1	0
	RP	M	85.0	91.5		0	
4	LP	F	82.0	92.0	89.5	0	2
	RP	F	85.5	92.5		0	
5	LP	M	82.5	91.0	92.0	1	1
	RP	M	85.0	88.5		2	
6	LP	F	73.0	82.5	85.5	3	1
	RP	F	83.0	93.5		2	
Mean			85.7	91.2	91.3	1.1	1.0

During the first session individual participants had to cover the full 105-cm range of potential ball arrival positions with their paddle initially positioned at an eccentricity of 21 cm (to the left for the LPs and to the right for the RPs) with respect to the center of the screen. With an average interception performance of 85.7 ± 5.4% for the total of 200 trials completed by each participant, performance was overall quite good. A repeated-measures one-way ANOVA on the evolution of performance over the four blocks of 50 trials revealed a significant effect of Block [*F*(3,33) = 18.51, *p* < 0.001, η^2^ = 0.63], reflecting an initial increase from Block 1 (78.2 ± 8.9%) to Block 2 (88.8 ± 4.2%), followed by a leveling off of performance during Blocks 3 (87.2 ± 6.3%) and 4 (88.7 ± 5.6%). *Post hoc* Newman–Keuls analyses confirmed that performance in Block 1 was significantly different from performance in Blocks 2, 3, and 4 (*p*’s < 0.001), while no significant differences were observed among the latter.

During the second session the individual participant’s paddle was initially positioned at an eccentricity of 30.25 cm (to the left for the LPs and to the right for the RPs) with respect to the center of the screen. Participants were assisted by a static partner (32-cm wide stationary paddle) covering the final 24-cm range of potential ball arrival positions on the opposite side of the full 105-cm range. They therefore needed to cover an 81-cm range of potential ball arrival positions while avoiding contact with the static partner’s paddle. Collisions with the stationary paddle occurred only sporadically (on average on 0.5 ± 0.6% of the trials, see **Table 1**), with only three participants colliding once during the first block. Interception scores were stable over blocks (89.3 ± 4.5, 91.7 ± 5.2, 93.0 ± 5.9, and 92.0 ± 4.5%, for blocks 1, 2, 3, and 4, respectively); a repeated-measures ANOVA did not reveal significant differences in performance over the four blocks [*F*(3,33) = 1.61, *p* = 0.205, η^2^ = 0.13]. These results indicate that participants performed well from the beginning of the session.

In order to examine potential differences between LPs and RPs in Sessions 1 and 2, we conducted a mixed two-way ANOVA on interception scores with Side (LP and RP) as a between-participant factor and Session (1 and 2) as a within-participant factor. This analysis did not reveal significant differences between LP and RP [*F*(1,10) = 1.22, *p* = 0.296, $\eta_{p}^{2}$ = 0.11]. Inspection of individual means (cf. **Table 1**) confirmed that performance was comparable for left and right participants in both sessions.

Having thus characterized the performance of individual participants in Sessions 1 and 2, we now turn to the third session in which the 12 participants were combined into six teams, each consisting of an LP and an RP. Paddles were initially positioned 30.25 cm to the left (LP) and to the right (RP) with respect to the center of the screen. Together, the two participants needed to cover the full 105-cm range of potential ball arrival positions while avoiding contact between their paddles. As in Session 2, collisions were rare (6 out of the total of 1200 trials, see **Table 1**), with only two teams colliding once within the first block. Interception scores were quite high from the start and stable over blocks (90.7 ± 4.3, 92.3 ± 3.9, 92.3 ± 5.1, and 89.7 ± 7.0%, for blocks 1, 2, 3, and 4, respectively); a repeated-measures one-way ANOVA did not reveal significant differences in performance over the four blocks [*F*(3,15) = 0.50, *p* = 0.688, η^2^ = 0.09]. Interestingly, team performance could not be predicted on the basis of its members’ scores observed in Session 2. Indeed in two cases team performance in Session 3 was better than the best team member’s score in Session 2 (teams 3 and 5, see **Table 1**). In two other cases the opposite pattern was observed (teams 1 and 4, see **Table 1**).

**FIGURE 4:**
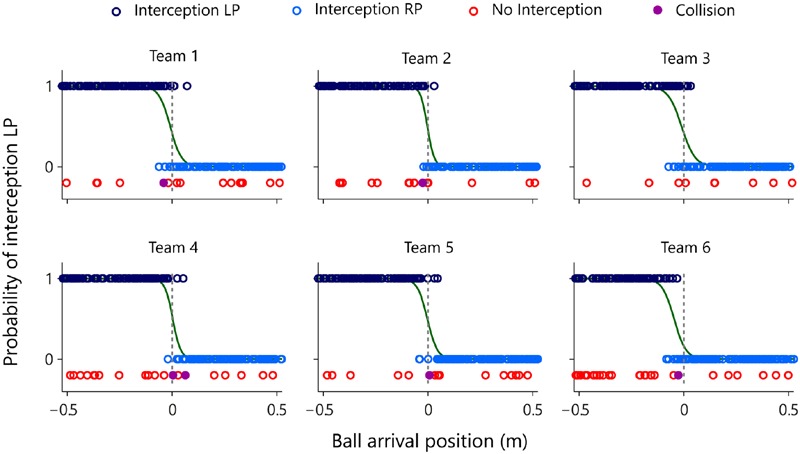
**Graphical summary of interception performance as a function of ball arrival position for all six teams separately.** Ball arrival positions for each successful trial are indicated by dark blue (LP interception) and light blue (RP interception) circles. Ball arrival positions of unsuccessful trials are indicated by red circles (errors) and purple dots (collisions). The green curves depict the logistic curves representing the probability that LP (*P* = 1) or RP (*P* = 0) will intercept the ball as a function of ball arrival position. The horizontal dashed gray lines at ball arrival position 0 cm indicate the center of the interception axis.

**Figure 4** provides a graphical summary of the interception results as a function of the ball’s arrival position on the interception axis for all 200 trials of each team. Interceptions accomplished by the LP (dark blue circles) and by the RP (light blue circles) were plotted on two separate axes, so as to allow visual discrimination of who intercepted the balls where. These intercepted trials were completed with the trials in which both participants failed to intercept the ball (red circles, referred to as errors) and with the trials in which the LP and RP paddles made contact with one another (purple dots, referred to as collisions). The (rare) collisions occurred for balls arriving at locations near the center of the screen. Errors, on the other hand, were generally distributed over the full range of ball arrival positions. Indeed, errors for ball arrival positions located within the interval between both participants’ initial positions (*n* = 53) occurred as often as errors for ball arrival positions outside this interval (*n* = 52), indicating that the majority of errors seemed to result from individual mistakes. Together with the high interception scores (on average 91.3 ± 3.4%) and the low number of collisions (on average 0.5 ± 0.3%), these results demonstrate that participants succeeded remarkably well in coordinating their interceptive movements with one another.

Visual inspection of **Figure 4** revealed that all six teams exhibited a quite well-defined distribution of who intercepted the ball where, with the LP intercepting the grand majority of balls arriving on the left half of the interception axis and the RP intercepting the grand majority of balls arriving on the right half. Interestingly, however, the interception performance of all teams also included an area where both participants could intercept balls. In order to quantify the separation of interception domains, for each team we computed a logistic regression equation with ball arrival position as the explanatory variable. Using a logit link function (Nelder and Wedderburn, 1972), logistic probability curves were derived for the balls intercepted by the LP (*P* = 1) and by the RP (*P* = 0) for all teams independently. The boundary between both interception domains was defined as the Median Effective Level (MEL), that is, the position on the interception axis where the probability of the LP intercepting the ball is equal to the probability of the RP intercepting the ball (i.e., *P* = 0.5). As can be seen from **Table 2** (observed interception performance), teams 1–5 revealed MEL values close to zero with a maximum absolute deviation of 1.08 cm, indicating that in these teams the boundary between both interception domains laid close to the exact (and yet unmarked) middle of the interception axis. Team 6, on the other hand, was characterized by a MEL value of -4.66 cm, indicating that the boundary between both interception domains was shifted almost 5 cm to the left. Of potential interest here is the fact that team 6 was the team with the largest difference in individual performances, as observed in Sessions 1 and 2 (see **Table 1**). The boundary shifted toward the participant with the lowest interception score, resulting in a 19.5% difference in the ranges of both participants’ interception domains. Note, however, that even in the presence of a shift in the location of the boundary team 6 still demonstrated a rather well-defined separation of interception domains.

**Table 2:** Logistic regression results for observed and predicted interception performance.

	Observed		AV Predicted	
Team	MEL (cm)	Overlap (cm)	MEL (cm)	Overlap (cm)
1	-0.88	16.1	-1.14	23.7
2	-0.31	9.7	-1.19	14.9
3	-1.08	19.3	-2.17	33.2
4	0.34	11.3	-1.27	20.9
5	-0.32	14.2	-0.25	15.1
6	-4.66	16.9	-4.24	22.0
Mean	-1.15	14.6	-1.71	21.6

The degree of separation between both interception domains is reflected in the steepness of the slopes of the logistic curve and the amount of overlap may be calculated as the distance between the 5 and 95% points of the logistic curve (Cox and Snell, 1989). On average, overlap thus defined amounted to a non-negligible 14.6 ± 3.6 cm. Interestingly, the amount of overlap between interception domains was not related to a team’s performance [*r* = 0.13, *t*(4) = 0.263, *p* > 0.8]. While team 6 (characterized by the leftward boundary shift discussed above) demonstrated an above-average overlap (16.9 cm, see **Table 2**) as well as the lowest team performance (85.5% of all balls intercepted, see **Table 1**), team 3 not only revealed the largest overlap (19.3 cm) but also the highest team performance (95.5% of all balls intercepted).

### Movement Kinematics

We first examined initiation times for all interception movements in all three sessions. As can be seen from **Table 3**, whereas average initiation times were similar for Sessions 1 (428 ± 38 ms) and 2 (437 ± 44 ms), they appeared longer for Session 3 (534 ± 51 ms). However, this observation was difficult to interpret because the different sets of initiation times refer to different ranges of movement in the three sessions. For Sessions 2 and 3 we therefore calculated the initiation times for the subset of all interception movements that were directed to ball arrival positions between the initial paddle position and the middle of the screen (i.e., between -30.25 cm and 0 cm for the LPs and between 0 cm and +30.25 cm for the RPs). As can be seen from the last two columns of **Table 3**, even for these range-corrected interception movements a difference in initiation time occurred [paired *t*-test: *t*(11) = 3.56, *p* < 0.01] with movements being initiated later in the presence of a dynamic partner (Session 3: 518 ± 50 ms) than in the presence of a static partner (Session 2: 459 ± 61 ms).

**Table 3:** Mean initiation times of individual participants in Sessions 1 (S1), 2 (S2), and 3 (S3).

			Initiation Times (ms)				
Team	Side	Gender	Full range			Range-corrected	
			S1	S2	S3	S2	S3
1	LP	M	409	477	581	479	645
	RP	M	404	344	492	371	487
2	LP	M	438	493	581	538	537
	RP	M	414	412	524	368	481
3	LP	M	412	423	483	421	518
	RP	M	462	462	499	467	540
4	LP	F	385	393	469	575	544
	RP	F	470	446	504	454	499
5	LP	M	364	393	508	478	513
	RP	M	451	474	556	406	449
6	LP	F	498	471	642	482	475
	RP	F	425	453	566	468	527
Mean			428	437	534	459	518

*Range-corrected initiation times only concern movements initiated for balls arriving between the initial position (-30.5 cm for the LP and +30.5 cm for the RP) and the center of the screen (0 cm).*

**FIGURE 5:**
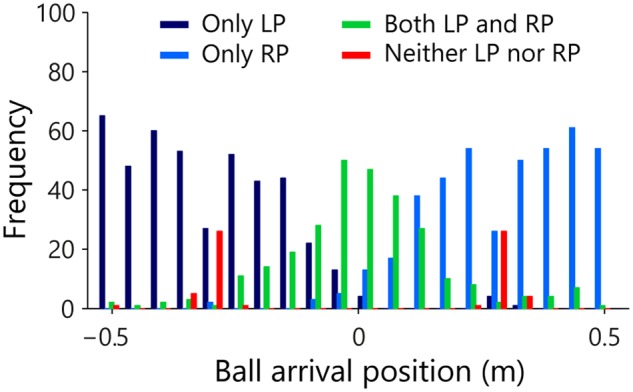
**Frequency distribution of the observed movement initiations of the LP and RP as a function of ball arrival position.** Each trial arriving in one of 20 (5.25-cm wide) bins was classified as indicating initiation of only LP (dark blue), only RP (light blue), both LP and RP (green) or neither LP nor RP (red).

In Session 3, interception on a given trial could *in fine* only be accomplished by a single participant but this did not necessarily imply that the other participant did not move at all. For every single trial and independent of the result, we therefore determined for both LP and RP whether they initiated a movement. **Figure 5** summarizes the resulting frequency distribution of observed movement initiations for the LPs and RPs as a function of the arrival position of the ball, with the full 105-cm range of potential ball arrival positions divided into 20 (5.25-cm wide) bins. Each trial was classified into one of four categories: initiation LP only (dark blue), initiation RP only (light blue), initiation both LP and RP (green), and no initiation, that is, neither LP nor RP (red) initiated a movement. Of all 1200 trials, 436 (i.e., 36.3%) revealed LP initiation only, almost exclusively associated with balls arriving on the left side of the interception axis. Similarly, 421 (i.e., 35.1%) of all trials revealed RP initiation only, almost exclusively associated with balls arriving on the right side of the interception axis. Of the 279 (i.e., 23.3% of all trials) revealing both LP and RP initiations, 246 (i.e., 88.2%) resulted in successful interception, implying that one of the participants must have abandoned the launched interception attempt at some point so as to allow the other participant to intercept the ball. The prevalence of such double initiations appeared to follow a bell-shaped distribution over the interception axis, with its peak located in the vicinity of the center of the interception axis (i.e., the center of the screen). In 5.3% of the trials neither of the two participants initiated any movement. In 63 of these 64 trials without movement initiation, balls arrived at or close to one of the participants’ initial positions (i.e., ±30.25 cm). Note that in 59 (i.e., 93.7%) of those 63 trials the ball was in fact intercepted, making contact with one of the motionless (3-cm wide) paddles.

**FIGURE 6:**
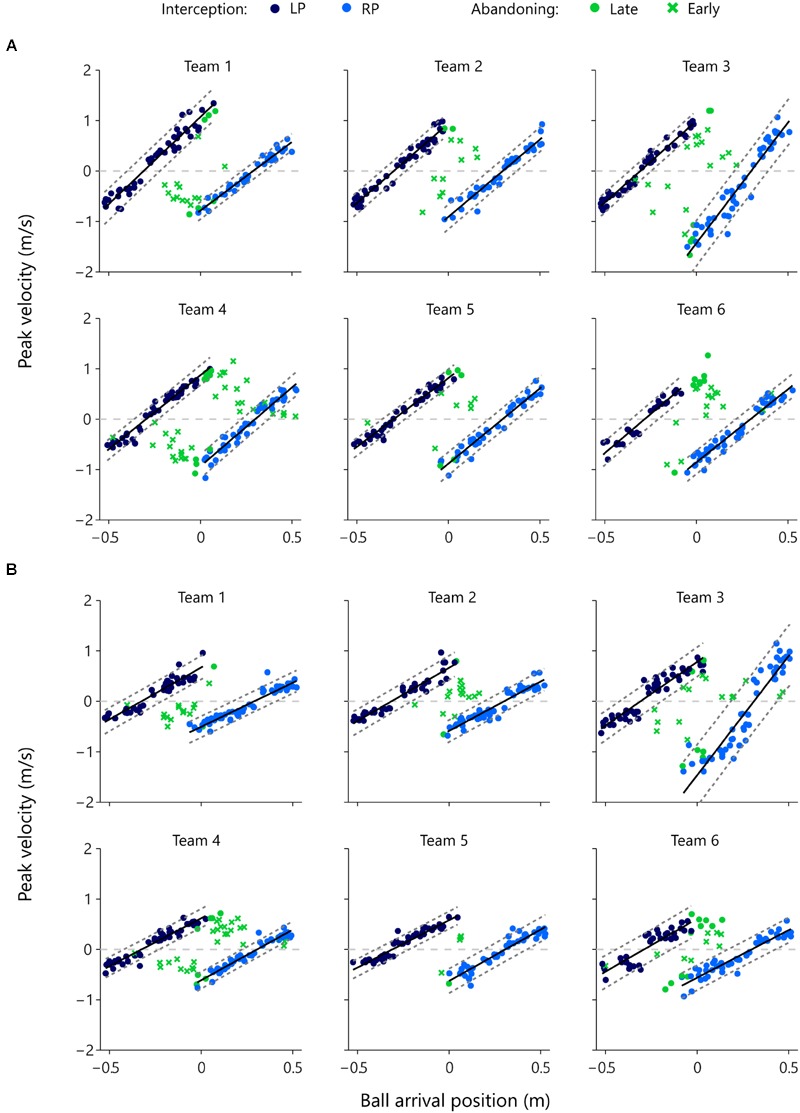
**Peak velocity of movement as a function of ball arrival position for both members of each team for each vertical ball speed separately.** Dark blue dots indicate LP-interception trials and light blue dots indicate RP-interception trials. The solid black lines represent the associated regression lines of peak velocity onto ball arrival position and the dashed gray lines represent the ±2 *SD* boundaries. Green symbols indicate trials in which interception was abandoned, with dots indicating that the peak velocity reached during that trial fell within the above-defined boundaries (late abandoning) and crosses indicating that the peak velocity reached during that trial fell outside the above-defined boundaries (early abandoning). The horizontal gray dashed lines in each panel, at peak velocity = 0 m/s, indicate the borders between negative (i.e., movements to the left) and positive (i.e., movements to the right) values of peak velocity. All green dots and crosses with positive peak velocity (i.e., all green points above the zero line) represent abandoned interception attempts of the LP. Likewise, all dots and crosses with negative peak velocity (i.e., all green points below the zero line) represent abandoned interception attempts of the RP. **(A)** High vertical ball speeds (0.64 m/s, 1-s trial duration) and **(B)** low vertical ball speeds (0.4 m/s, 1.6 s trial duration).

In order to obtain a grasp on when one of the participants abandoned the launched interception attempt, we examined the relation between the distance to be covered and the peak velocity reached during the movement on each trial. **Figure 6** presents this relation for each successful (i.e., intercepted) trial in which at least one participant initiated a movement, for each team and each of the two vertical ball speeds separately. Successful interceptions by the LPs (dark blue dots) and the RPs (light blue dots) were characterized by proportional scaling relations between the distance covered (i.e., the distance between initial paddle position and ball arrival position) and the peak velocity reached during the movement (see Ledouit et al., 2013, for similar results). For each individual player we therefore performed a linear regression analysis of peak velocity onto distance covered for the balls intercepted by that participant. Results of these regression analyses are reported in **Table 4** and shown graphically in **Figure 6**.

**Table 4:** Results of regression analyses of the relations between peak velocity and distance covered during movements resulting in interception, performed for each participant separately for each of the two vertical ball speeds.

			High Ball Speed				Low Ball Speed			
Team	Side	Gender	*n*	*a*	*r*	*p*	*n*	*a*	*r*	*p*
1	LP	M	43	3.46	0.97	<0.001	41	2.06	0.94	<0.001
	RP	M	39	2.73	0.98	<0.001	51	1.75	0.95	<0.001
2	LP	M	45	3.16	0.98	<0.001	39	2.12	0.96	<0.001
	RP	M	41	3.08	0.97	<0.001	50	1.97	0.95	<0.001
3	LP	M	42	3.25	0.98	<0.001	49	2.53	0.95	<0.001
	RP	M	43	4.80	0.96	<0.001	46	4.74	0.94	<0.001
4	LP	F	42	2.94	0.98	<0.001	46	1.90	0.96	<0.001
	RP	F	41	3.13	0.97	<0.001	43	2.01	0.97	<0.001
5	LP	M	41	2.74	0.98	<0.001	49	1.88	0.96	<0.001
	RP	M	39	2.99	0.98	<0.001	45	2.04	0.94	<0.001
6	LP	F	25	3.09	0.97	<0.001	35	2.10	0.89	<0.001
	RP	F	49	2.90	0.97	<0.001	46	1.89	0.94	<0.001

*n, number of trials; a, slope (s^-1^); r, correlation coefficient; p, probability.*

While the slope of the relation varied both as a function of participant characteristics and as a function of vertical ball speed, individual correlation coefficients were satisfactorily high to allow the definition, for each participant at each vertical ball speed, of a “standard” relation (operationally defined by a range of ±2 *SD*s around the mean, dashed parallel lines in the panels of **Figure 6**) between ball arrival position and peak velocity reached during an interception movement. Using this “standard” relation observed for successful interceptions, we could identify whether the 246 abandoned interception attempts (i.e., successful trials in which the participant that did not intercept the ball had nevertheless initiated a movement) occurred early or late during the trial. Late abandoning was characterized by the participant still reaching the standard peak velocity (green dots in **Figure 6**), whereas early abandoning was characterized by the participant reaching a lower-than-standard peak velocity (green crosses in **Figure 6**). Of the 246 successfully intercepted trials demonstrating both LP and RP initiation, 179 (i.e., 72.8%) were characterized by early abandoning, while 67 (i.e., 27.2%) were characterized by late abandoning.

### Team Interactions

Several of the results discussed in the previous sections suggest that team performance, as observed in Session 3, cannot be satisfactorily understood as resulting from a form of organization with pairs of independent players, each covering their own half of the interception space. First, while for five of the teams the boundary between interception domains laid close to the center of the screen (with differences in the sizes of individual participant interception domains being limited to 2.3 ± 1.4%), in team 6 this boundary was shifted by almost 5 cm, leading to a difference in domain sizes of 19.5%. Second, for all six teams the boundary between interception domains was fuzzy rather than sharp, with participants regularly entering their teammate’s domain to intercept balls there without such “intrusions” leading to collisions. The observed degree of overlap between interception domains was indeed quite substantial (14.6 ± 3.6 cm), amounting to 13.9 ± 3.4% of the full range of potential ball arrival positions. Third, balls arriving near the center of the screen (four center bins of **Figure 5**, with ball arrival positions ranging from -10.5 to +10.5 cm) more often evoked movement initiations of both participants than only initiations of the participant in whose interception domain the ball would in fact arrive. Yet, both collisions and errors were rare, as 87.9% of the trials on which both participants initiated a movement resulted in successful interception by one of the participants. Finally, while in 72.8% of the 246 double-initiation trials one of the participants abandoned the launched interception attempt early on, in the remaining 27.2% of the trials the interception attempt was abandoned after the participant had reached a peak velocity associated with an ongoing interception attempt. Together, these results suggest that participants took into account the ongoing actions of their partners.

Without going as far as suggesting that this is the information used by the participants (see Fajen and Warren, 2007; Bootsma et al., 2016, for further details), for the present purposes the state of the angle formed, for each participant, by the line connecting this participant’s paddle with the other participant’s paddle and the line connecting this participant’s paddle with the ball (see **Figure 3**) may well allow capturing the unfolding team interactions. Indeed, by physical law, a constant angle (i.e., a zero AV) indicates that the player’s current movement speed will lead the paddle to reach the interception point when the ball arrives there. Put differently, zero AV means that an interception will occur if both ball and paddle speed remain constant over the remainder of the trial. Given that in the present study ball speed was always constant over the course of a trial, from the foregoing it follows that a positive AV (i.e., an opening of the angle) implies that maintaining current paddle speed will lead to an early arrival at the interception location and, likewise, that a negative AV (i.e., a closing of the angle) implies that maintaining current paddle speed will lead to a late arrival at the interception location.

When neither of the two participants has begun to move their paddle (i.e., from the beginning of a trial up to the moment of first movement initiation), for both participants AV will be negative for balls arriving at a location between the two paddles. For balls arriving at locations to the left of the LP, AV will be positive for the stationary LP and negative for the stationary RP. *Mutatis mutandis*, AV will be positive for the stationary RP and negative for the stationary LP for balls arriving at locations to the right of the RP.

**FIGURE 7:**
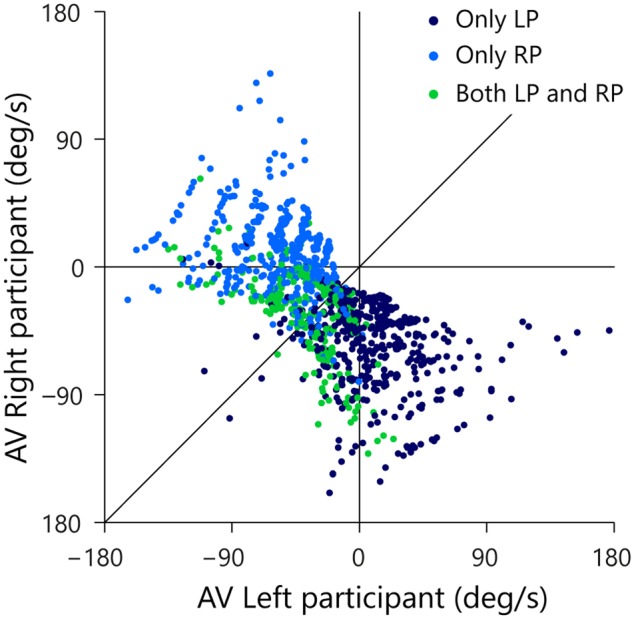
**Rate of change of β_RP_ (AV-RP) as a function of rate of change of β_LP_ (AV-LP) at the moment of first participant movement initiation.** Trials with only LP initiation are indicated by dark blue dots, trials with only RP initiation by light blue dots, and trials with both LP and RP initiation by green dots. The thin vertical and horizontal lines mark zero AV for the LP and RP, respectively. The thin diagonal line marks AV-LP = AV-RP.

Each trial in which one or both participants initiated a movement is represented in **Figure 7** as a point in space defined by the states of the AV-LP (abscissa) and the AV-RP (ordinate) at the moment of first movement initiation. Dark blue dots designate the 436 trials in which only the LP initiated a movement, light blue dots designate the 421 trials in which only the RP initiated a movement, and green dots designate the 279 trials in which both players initiated a movement. As was already visible in **Figure 5**, balls arriving to the left of the LP almost invariably evoked only movement from the LP. In **Figure 7**, these trials correspond to the (predominantly dark blue) dots in the lower right quadrant where AV-LP is positive and AV-RP is negative. Likewise, balls arriving to the right of the RP almost invariably evoked only movement from the RP. In **Figure 7**, these trials correspond to the (predominantly light blue) dots in the upper-left quadrant where AV-LP is negative and AV-RP is positive. As was also already visible in **Figure 5**, trials evoking initiation by both the LP and RP generally arrived between the initial positions of both paddles, close to the center of the screen. In **Figure 7** these trials correspond to the green dots predominantly located in the lower-left quadrant where both AV-LP and AV-RP are negative.

**FIGURE 8:**
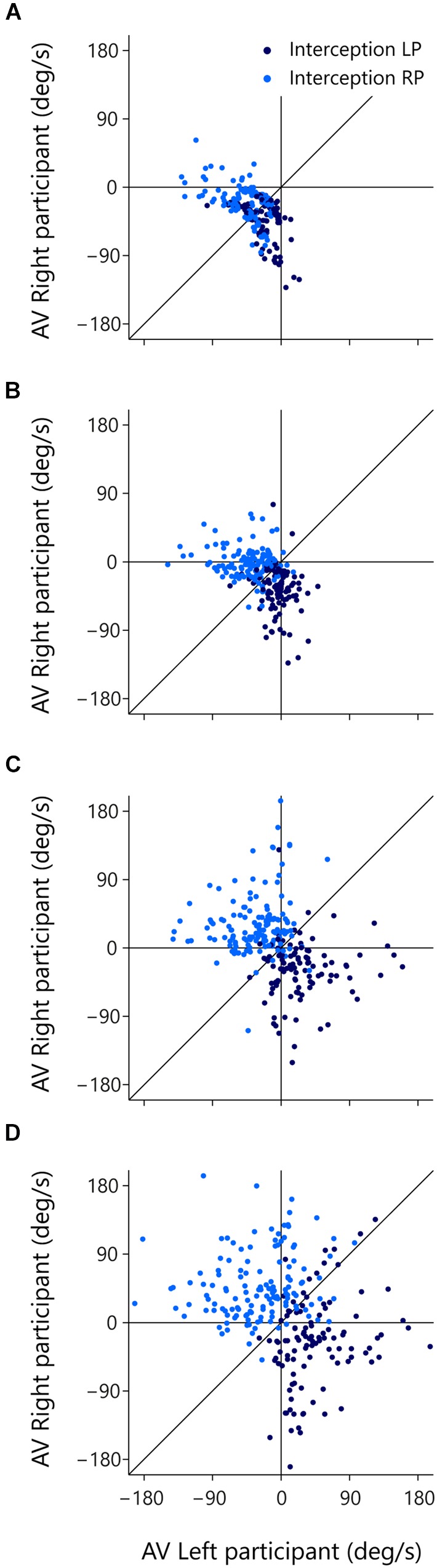
**Rate of change of β_RP_ (AV-RP) as a function of rate of change of β_LP_ (AV-LP) for the trials with both LP and RP initiation at different moments in time.** Dark blue dots indicate LP-interception trials and light blue dots indicate RP-interception trials. The thin vertical and horizontal lines in each panel mark zero AV for the LP and RP, respectively. Movement of dots across these lines mark transitions from negative to positive AV. The thin diagonal line in each panel marks AV-LP = AV-RP. **(A)** At the moment the first participant initiated a movement, **(B)** 100 ms later, **(C)** 200 ms later, and **(D)** 300 ms later.

The (AV-LP, AV-RP) state space allows us to scrutinize the evolution over time of the behavior of both participants with respect to the ball. The trials of interest for such scrutiny are of course the trials in which both participants initiated an interception movement (green dots in **Figure 7**). For these reasons, the subset of 246 successfully intercepted trials in which both participants initiated a movement is once again presented in **Figure 8**, but this time coded for the player who in the end intercepted the ball (LP interception: dark blue, RP interception: light blue). When participants start moving they actively change their relation to the ball, which is functionally captured by a change in their AV. The motion through the (AV-LP, AV-RP) state space thus captures the dynamic triangular relation between both players and the ball. As in **Figure 7**, Figure **8A** depicts the situation at the time of first movement initiation. **Figures 8B–D** depict the situation, respectively, 100, 200, and 300 ms later.

Inspection of **Figure 8** brings out that trials that eventually gave rise to LP-interception were characterized by a change in AV-LP from negative to positive (resulting from the LP’s sustained movement toward the future interception location), with dots moving from the lower-left quadrant either to the lower-right quadrant or, for a smaller proportion of trials, to the upper-right quadrant. A similar picture emerged for the trials that eventually gave rise to RP-interception. These trials were characterized by a change in AV-RP from negative to positive (resulting from the RP’s sustained movement toward the future interception location), with dots moving from the lower-left quadrant either to the upper-left quadrant or, for a smaller proportion of trials, to the upper-right quadrant. **Figure 8** thus reveals the gradual separation in the two groups of trials based on who intercepted the ball in the end. This observation suggests that the decision of who intercepts the ball in fact emerges over the course of a trial, as a function of the expediency with which both participants engaged in their interception attempts. In fact, it appeared that the first participant to reach positive AV tended to be the one that ended up intercepting the ball. Recalling (cf. **Figure 3**) that negative AV implies that with the current movement speed the participant will be (too) late, positive AV implies that with the current movement speed the participant will in fact arrive at the interception location before the ball gets there. Even though all participants generally slowed down prior to interception (probably so as to minimize chances of colliding with the other participant), the occurrence of a positive AV for one participant may signal to the other that the interception attempt should be abandoned.

**FIGURE 9:**
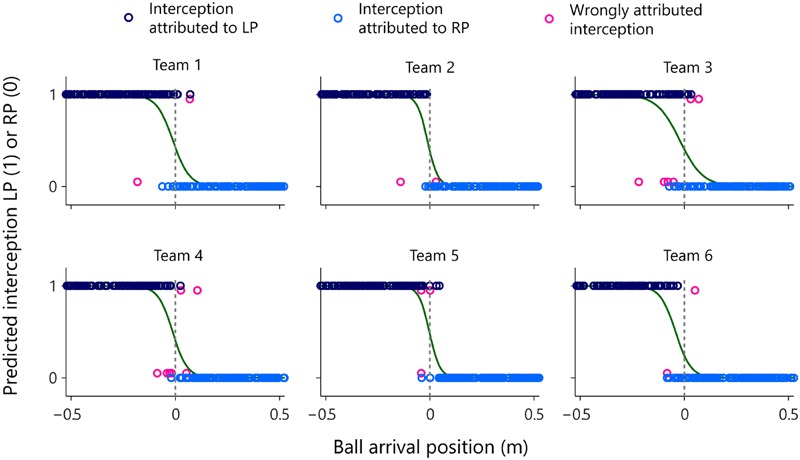
**Graphical summary of predicted interception performance as a function of ball arrival position for all six teams separately.** The participant that would intercept the ball was predicted as being the participant who first reached positive AV. Ball arrival positions for correctly attributed interceptions are indicated by dark blue (LP interception) and light blue (RP interception) circles. Ball arrival positions of incorrectly attributed interceptions are indicated by pink circles with a slight vertical offset. The green curves depict the logistic curves representing the probability that LP (*P* = 1) or RP (*P* = 0) will intercept the ball as a function of ball arrival position. The horizontal dashed gray lines at ball arrival position 0 cm indicate the center of the interception axis.

In order to test this idea, we examined the evolution over time of AV-LP and AV-RP for all 1095 trials on which the ball was intercepted. Starting from the situation at the onset of a trial, we classified the trial as LP-interception or RP-interception, as a function of the first participant to reach positive AV. Note that this rule led to correct (although immediate) classification of balls arriving to the left of the LP as LP-interception and of balls arriving to the right of the RP as RP-interception. The results of this on-the-fly decision formulation are presented in **Figure 9** for all six teams separately.

As can be seen from **Figure 9**, attribution of interception to the LP (dark blue circles) or the RP (light blue circles) was correct in the overwhelming majority of cases. Overall, attribution errors occurred on only 2.0% of the trials, corresponding to a total number of errors of 2, 2, 6, 7, 3, and 2, for teams 1–6, respectively. The on-the-fly decision criterion of interception by the “first participant to reach positive AV” not only allowed to predict which participant would intercept the ball with more than satisfactory precision, but also reproduced the qualitative aspects of the distribution of interception domains observed in each team. Deriving logistic probability curves for the predicted performance (see **Table 2**, predicted interception performance) revealed that the locations of boundaries between interception domains were well predicted [*r* = 0.92, *t*(4) = 4.54, *p* = 0.010], laying close to the center of the screen (2.17 cm maximal absolute deviation) for teams 1–5 while being shifted 4.24 cm to the left for team 6. Similarly, even though somewhat overestimated, the amount of overlap between interception domains was fairly well predicted [*r* = 0.80, *t*(4) = 2.63, *p* = 0.059].

Finally, because the moment at which the first participant reached positive AV could be detected, we examined whether this criterion also correctly predicted when the non-intercepting participant abandoned the launched interception attempt in the trials in which both participants initiated an interception movement. In 209 (i.e., 85.0%) of the 246 double-initiation trials, the abandoning participant indeed reached peak velocity *after* the intercepting player had reached positive AV. Thus, the non-intercepting participant was already decelerating (that is, had already abandoned) before the intercepting player reached positive AV in only 15.0% of the cases. This first analysis suggests that our on-the-fly decision criterion also captures the timing of the decision rather well. We can take the analysis one step further by also considering the information with respect to the moment of abandoning contained in the magnitude of the peak velocity reached by the non-intercepting participant, as described in Section “Movement Kinematics.” If the peak velocity reached during an abandoned interception attempt corresponded to the “standard” peak velocity of a successful interception movement, the interception attempt was considered as still underway at the moment the non-intercepting participant reached this peak velocity. Abandoning was then classified as late. If, on the other hand, the peak velocity reached during an abandoned interception attempt was smaller than the standard peak velocity, the interception attempt was considered as already abandoned when the non-intercepting participant reached this lower-than-standard peak velocity. Abandoning was then classified as early. **Table 5** presents the foregoing results in the form of a contingency table.

**Table 5:** Contingency table for double-initiation (both LP and RP) trials, combining the number of times the non-intercepting player reached peak velocity before or after the intercepting participant reached positive angular velocity with the number of times the non-intercepting player abandoned the interception attempt early or late, as determined by the magnitude of the peak velocity reached.

	Before	After	Total
Early	29	150	179
Late	8	59	67
Total	37	209	246

As can be seen from **Table 5**, of the 209 double-initiation trials in which the non-intercepting participant reached peak velocity *after* the intercepting participant had reached positive AV, 150 (i.e., 71.8%) had been characterized as early abandoning and 59 (i.e., 28.2%) as late abandoning. This repartition nicely mirrors the observed overall 72.8% (179 out of 246) early abandoning and 27.2% (67 out of 246) late abandoning. Of the 37 trials in which the non-intercepting participant had reached peak velocity *before* the intercepting participant reached positive AV, the grand majority (29 or 78.4%) had been characterized as early abandoning. We suggest that in many of these trials the non-intercepting participant produced only a small movement, characterized by a low peak velocity (i.e., the green points close to the zero velocity axis in **Figure 6**). Overall we conclude that the on-the-fly criterion that the ball will be intercepted by the “first participant to reach positive AV” allows the observed team interactions to be rather accurately captured.

## General Discussion

In the present contribution we set out to study how a team of two players coordinated their actions so as to intercept a series of approaching balls. Contrary to most work performed in the field of between-participant collaboration (e.g., Mottet et al., 2001; Isenhower et al., 2010; Romero et al., 2015), our doubles-pong task (implicitly) required the team members to decide amongst them, on every single trial, who would perform the interceptive action and who would not: continuing interception attempts realized by both players led to collisions between their paddles that subsequently disintegrated, thereby no longer allowing the ball to be intercepted. In order to be able to study how such joint decisions were made on the basis of shared visual information only, we effectively prevented participants from directly communicating between them: unable to see or hear the other participant, they only shared the visual information available on the screen in front of them, depicting the moving ball and the positions of each of the two participant-controlled paddles along the interception axis.

Before partaking in the team interception session, participants had previously been familiarized with the apparatus and task. In a first session they had practiced intercepting all balls on their own and in a second session they had practiced intercepting balls while assisted by a static partner, incorporated by a large stationary paddle covering the last part of the opposite side of the interception axis. These first two sessions not only served to allow the participants to become acquainted with the set-up but also allowed us to characterize interception performance of all 12 individual participants. After having ascertained that performance in the first two sessions was comparable for the left-positioned participants (LPs) and right-positioned participants (RPs), six teams, each consisting of an LP and a RP, were formed for the final session.

Notwithstanding the lack of possibilities for overt communication, performance during this team interception session was remarkably good, with between 85.5 and 95.5% of the balls being intercepted by the different teams. Collisions were extremely rare, with one team never colliding, four teams colliding once and one team colliding twice on a total of 200 trials per team. Focusing on who intercepted balls where revealed that all teams instantiated a division of the total interception space, with the LP intercepting the grand majority of ball arriving on the left half of the interception axis and the RP intercepting the grand majority of balls arriving on the right half. However, as already mentioned, a simple geometry-based division-of-space hypothesis did not satisfactorily account for the pattern of results observed. The decision of who intercepts a ball where appeared to be founded in between-participant interactions rather than in situational geometry.

A first indication hereof was the finding that, while for five of the six teams the boundary between LP and RP interception domains was located close to the (unmarked) center of the screen, for the remaining team this boundary was shifted almost 5 cm to the left (cf. **Figure 4**). As the latter team was characterized by a large difference in individual performance scores in Sessions 1 and 2 and the LP was the participant with the lowest interception performance scores, it is tempting to suggest that the boundary shift resulted from the better (worse) player taking charge of a larger (smaller) part of the interception space. However, more systematic explorations of between-participant performance levels are required to test the hypothesis that a team’s division of interception space may indeed depend on the performance levels of the individual members. By the same token, the question whether approximately equally skilled team members would also divide the interception space in halves when their initial paddle positions were not symmetrically centered around the middle of the space also needs to be addressed in future work.

A second indication of the inadequacy of a geometry-based division-of-space hypothesis was the finding that, even though all six teams of the present study revealed a division of interception space, such divisions were never absolute. Boundaries were indeed fuzzy rather than clear-cut and the interception domains of individual participants were characterized by a significant degree of overlap (cf. **Figure 4**). Under a division-of-space hypothesis excursions into the other participant’s interception space should be considered as mistakes likely to result in collisions, with the likelihood of collisions expected to increase with the magnitude of the intrusion. Yet excursions into the partner’s interception domain leading to successful interception were clearly far more frequent than collisions. Collisions moreover generally occurred for balls arriving very close to the boundary between interception domains. Interestingly, overlap between interception domains was not only spatial but also temporal: initiation of interceptive movements by both participants occurred in almost a quarter of all trials (cf. **Figure 5**). While this may be understood as resulting from uncertainty with respect to the future ball arrival position, it does require that at some point in time one of the participants abandons the launched interception attempt so as to allow the other participant to successfully intercept the ball. At least in these trials the decision to (continue to attempt to) intercept the ball on a given trial or not is thus clearly taken on the fly rather than before movement onset.

How might between-participant interactions provide an account for the patterns of results observed? In the present contribution we suggested that the dynamic triangular relations between the movements of both participants and the approaching ball may be captured by the relation between the rates of change of angles β_LP_ and β_RP_ (cf. **Figure 3A**). Importantly, both AVs are influenced by the motion of the ball. Moreover, AV-LP is influenced by the way in which the LP moves the left paddle and AV-RP is influenced by the way the RP moves the right paddle. Contrary to movement speed, that necessarily varies as a function of the distance to be covered, AV provides a functional (because future outcome-related) characterization of the relation between the ball and the participant’s paddle (see **Figures 3B–D**). As such it allows evaluation of the expediency of both participants’ ongoing interception attempt. Expediency here refers to the current functionality of the engagement of a participant in an interception attempt, with an expedient movement being a movement that rapidly leads to positive AV. Because positive AV implies a paddle speed that is higher than required to ensure interception, such a relation indicates that the participant is on track to perform an interception (and may end up beyond the interception point if the ongoing movement is not decelerated). Picking up such expediency of the partner’s movement would allow the other participant to timely abandon his/her own ongoing interception attempt in order to avoid the paddles to collide.

Simulating the outcome of the on-the-fly decision process on each intercepted trial by attributing the future interception to the first participant to attain positive AV allowed the qualitative aspects of the observed results to emerge for all six teams. Indeed the predictions grounded in this action-based criterion (cf. **Figure 9**) revealed that the overlap as well as the location of the boundary between interception domains, including the boundary shift observed for team 6, could be understood as emerging from the participants’ behaviors during a trial. It is worth noting that predicted overlap tended to be larger than observed overlap, emphasizing the capacity of an information-based coupling to explain such a phenomenon. Moreover, the simulation provided first evidence that not only the outcome but also the timing of the team’s decision who will intercept the ball could be understood as emerging from the interaction.

In this study we took an embodied approach to joint decision making (Richardson et al., 2008; Marsh et al., 2009; Richardson et al., 2010; Coey et al., 2012). Looking at the interactive team behavior over time provides a way to study the emerging of the decision over time, rather than focusing on the outcome of a decision making process (cf. Turvey and Shaw, 1995; Lepora and Pezzulo, 2015). With the observation that in almost a quarter of all trials both participants initiated an interceptive movement (after which one of the two was required to abandon this attempt), the results of the present study provide behavior-based empirical evidence for the argument that actions may already be underway before decisions are completed, stressing the need to consider choice of action and control of action as highly integrated rather than serially arranged processes (e.g., Newell and Simon, 1972; for neural accounts also proposing parallel rather than serial decision processes, see, for instance, Cisek, 2007; Lepora and Pezzulo, 2015). The results also revealed that team decisions do not necessarily call upon shared knowledge or mental models —minimally exemplified in our doubles-pong task without overt communication by a silent agreement to divide interception space— as suggested by tenants of the social-cognitive perspective (e.g., Cannon-Bowers et al., 1993; Eccles and Tenenbaum, 2004; Cannon-Bowers and Bowers, 2006; Ward and Eccles, 2006; Sebanz and Knoblich, 2009). Our results rather suggest that team decisions are information-driven: the interactions between the participants with respect to the ball provide information (tentatively captured in the AV-LP, AV-RP space) that can be used to decide to continue or to abandon a launched interception attempt.

Taking our observations into account, how then should we perceive a team of two individuals intercepting balls together? Intercepting a moving target on itself is a non-social activity and, therefore, often studied as such (e.g., Bootsma and Van Wieringen, 1990; Peper et al., 1994; Chardenon et al., 2005; Michaels et al., 2006; Fajen and Warren, 2007; Ledouit et al., 2013; Bootsma et al., 2016). However, whereas the ball typically will be intercepted by one individual, in many (sports) situations more individuals are present, potentially intercepting the ball as well. The task under study here was inspired by and modeled after the situation of (beach) volleyball players ready to intercept an oncoming serve. In situations such as these, it is the common goal (i.e., intercepting as many balls as possible) and accompanying constraints (i.e., not colliding with one another) that bind both individuals to act as a ‘social unit’ (i.e., a team; Marsh et al., 2006). Nevertheless, we do not know (yet) how such a social unit comes about from two ‘I’s’ cooperating as a ‘we’ on the same task. Marsh et al. (2006) proposed that multiple individuals acting together might be considered a so-called social synergy, in which several individuals are temporally and functionally constrained by informational linkages to act as one unit. Evidence for such a synergistic approach to joint action has been found in studies on rhythmical interpersonal coordination (see Schmidt and Richardson, 2008 for a review) and during a continuous interpersonal postural task (Ramenzoni et al., 2011) showing behavioral control at the collective level. Our study, however, does not concern continuous rhythmical movements made by an ensemble of individuals, neither do both individuals perform the same task, as only one of the two individuals will intercept the ball in the end. Our results, though, do suggest that both players act as a team when deciding to go for the ball or not.

## Conclusion

This study offered a paradigm in which two players act as a team to realize the interception of an approaching ball without any other means of interaction than the visual information of the joint action display on the shared task space. We suggest that the decision of who of the two players realizes ball contact emerges from these interactions of both players (paddles) and the ball. The coordinated action often involves the initiation of movement by both members of a team, leading to abandoning of movement by one of the players. Of course, many questions remain. Details of the interactions, effects of the means of interacting, and the identification of the information that the players use await future experiments. Furthermore, we suggest that the task that we developed captures the essentials of real-world tasks such as the interception of a serve in beach volleyball, but also in many other situations of daily life in which individuals have to coordinate to attain a common goal. Although further testing is needed to back up these suggestions, we feel that the paradigm that we introduced holds great promise for understanding on-the-fly decision making among individuals.

## Author Contributions

NHB, FZ, RC, NB, and RB conceived and designed the experiments and critically contributed to the intellectual content of the work. RC conceived the experimental set-up. NHB ran the experiments. NHB, FZ, RC, and RB analyzed the data. NHB, FZ, and RB wrote the first drafts. All authors approved the final version of the manuscript.

## Conflict of Interest Statement

The authors declare that the research was conducted in the absence of any commercial or financial relationships that could be construed as a potential conflict of interest.
